# Exploring Chinese Millennials’ Minimalist Consumption Behavior in the Context of Global Economic Downturn: A PLS-SEM Analysis Based on Symbolic and Simulacrum Consumption Perception

**DOI:** 10.3390/bs14121175

**Published:** 2024-12-08

**Authors:** Ya Lu, Tian Zhang, Zhiguo Xu

**Affiliations:** 1 School of Marxism, Nanjing University of Science and Technology, Nanjing 210094, China; luyalook@njust.edu.cn; 2 School of Public Affairs, Nanjing University of Science and Technology, Nanjing 210094, China; zhangtian_psy@njust.edu.cn

**Keywords:** millennials, consumer behavior, symbolic consumption perception, simulacrum consumption perception, minimalist consumption behavior, upward social comparison

## Abstract

In the context of a global economic downturn, Millennials—who represent the emerging primary consumer demographic—are increasingly adopting a minimalist consumption model. To understand this phenomenon, this study employed a partial least squares structural equation modeling (PLS-SEM) analysis of 554 survey responses from Chinese Millennials. Building on the Theory of Reasoned Action, we explored the effects of consumption orientation, perceived economic pressure, self-expression, and perceived consumption risk on symbolic and simulacrum consumption perceptions. Furthermore, we investigated how these perceptions shape minimalist consumption awareness and behavior. The findings indicate the following: (1) consumption orientation significantly and positively influences symbolic consumption perception; (2) self-expression and perceived consumption risk positively affect both symbolic and simulacrum consumption perceptions; (3) symbolic consumption perception has a positive impact on minimalist consumption awareness; and (4) upward social comparison exerts a significant negative moderating effect on the relationship between minimalist consumption awareness and behavior. This study is the first to integrate symbolic and simulacrum consumption into the analytical framework of Millennials’ minimalist consumption behavior, shedding light on the nuanced relationship between self-expression and consumption behavior under economic pressure. The findings contribute a novel theoretical perspective for future research on minimalist consumption and offer practical insights for businesses aiming to devise effective marketing strategies amidst economic challenges.

## 1. Introduction

The World Bank projects that the global economy will grow by 2.2% from 2020 to 2024, marking the weakest five-year growth rate since the 1990s [1]. Against the backdrop of slowing global economic growth and downward pressures on the Chinese economy, Millennials, as the main consumer force, play a crucial role in driving domestic demand and promoting economic growth through their shifts in consumer behavior. Therefore, a thorough exploration of minimalist consumption behavior among Millennials holds significant practical importance for interpreting consumption trends in the current economic environment.

“Millennials” typically refers to individuals born between the early 1980s and late 1990s, i.e., those born between 1980 and 1999 [2]. Millennials are regarded as the most “distinctive” generation, having witnessed the transition from analog to digital and, crucially, having grown up amidst the proliferation of the internet, smartphones, and social media. They have experienced economic prosperity brought about by accelerated globalization and, during financial crises like the 2008 global financial crisis, have profoundly felt economic uncertainty [3,4]. However, it is precisely this “distinctive” generation that now constitutes the most potent consumer force, with their spending habits directly influencing national and even global economic trends [5]. Previous research has shown that, compared to other generations, Millennials tend to have a stronger inclination towards purchasing products with strong symbolic meaning and simulacrum value, such as luxury handbags and virtual game credits [6,7]. In recent years, economic downturns have fueled the rise of “minimalism,” with the concepts of “decluttering and minimalism” gradually penetrating the lives of Millennials. Facing economic uncertainty, employment pressures, and rising living costs, this generation has begun to reassess their consumption patterns, gradually shifting from consumerism to minimalist consumption that emphasizes intrinsic value. However, the transformation in Millennials’ consumer behavior is not merely an economic phenomenon; it is also profoundly influenced by psychological and social factors. Symbolic consumption perception and simulacrum consumption perception, as crucial consumption characteristics, reveal consumers’ perception and pursuit of the additional social value of goods. Against the backdrop of economic downturns, how Millennials recognize the symbolic and simulacrum features of consumption through psychological perception and social cognition, which thereby strengthens their minimalist consumption consciousness and further translates it into minimalist consumption behavior, has become an important issue that urgently needs to be explored.

Although existing research has focused on Millennials’ consumer behavior and intergenerational characteristics, there is currently limited systematic discussion combining symbolic consumption, simulacrum consumption, and minimalist consumption behavior [8,9]. Using the Theory of Reasoned Action as a framework, this study analyzes multiple factors that may influence symbolic and simulacrum consumption perceptions, discusses how these two consumption perceptions affect minimalist consumption consciousness, and examines the process of transforming minimalist consumption consciousness into behavior. The Theory of Reasoned Action suggests that individuals make choices in the behavioral decision-making process based on expectations of behavioral outcomes, attitudes, social norms, and behavioral intentions. With this theoretical framework, this study’s analysis considers the following four key dimensions. Consumption orientation is a significant factor influencing symbolic and simulacrum consumption perceptions, reflecting consumers’ focus on the symbolic meaning and simulacrum value behind goods, transcending their actual functional value. Meanwhile, analysis of perceived economic pressures, as an external environmental factor, can reveal how economic downturns intensify consumers’ cognitive need for symbolic and simulacrum consumption. In addition, self-expression plays a crucial role in consumer behavior, and an enhancement in self-expression ability prompts consumers to choose products they genuinely need rather than homogenized symbolic or simulacrum products. Finally, perceived consumption risk reflects consumers’ weighing of potential benefits and risks in decision making regarding symbolic or simulacrum consumption. These variables collectively construct the causal pathway between consumers’ psychological perceptions and behavioral decisions. To further uncover the role of social factors, this study introduces social comparison as a moderating variable, exploring how comparison with others (especially those in higher social strata) influences consumers’ transformation from minimalist consumption consciousness to minimalist consumption behavior.

Based on these concepts, this paper employs PLS-SEM methodology to construct and validate a comprehensive framework using data from 554 questionnaires completed by Chinese Millennials, exploring how symbolic consumption perception and simulacrum consumption perception drive the formation of minimalist consumption behavior against the backdrop of economic downturns. The findings reveal the pivotal role of psychological factors in converting economic and social pressures into behavioral changes, with a focus on genuine needs rather than excessive consumption, thereby expanding the application of the Theory of Reasoned Action in the field of consumer behavior. Meanwhile, this study provides practical suggestions for businesses to implement value-oriented consumption strategies, helping to attract Millennial consumers who prioritize practicality, and offers significant insights for policymakers in designing sustainable consumption policies.

## 2. Literature Review and Research Hypotheses

### 2.1. Literature Review

#### 2.1.1. Theory of Reasoned Action Model

**Figure 1:**
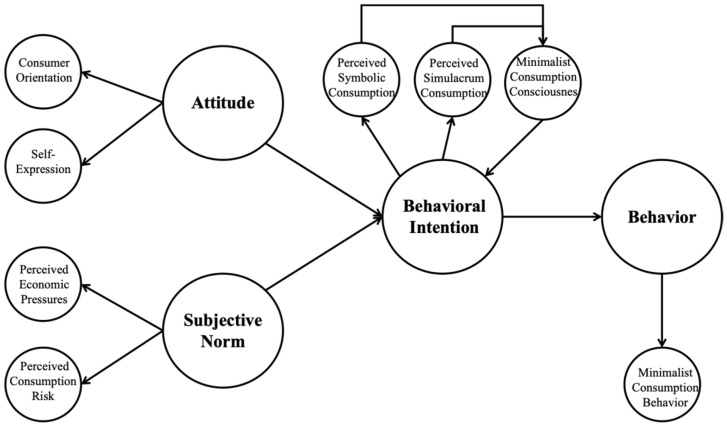
Theory of Reasoned Action model.

The Theory of Reasoned Action (TRA), proposed by Ajzen and Fishbein, is a theoretical framework widely used in the fields of social psychology and behavioral science. It is mainly used to explain and predict an individual’s behavioral intentions and actual behaviors [10]. The core idea of this theory is that an individual’s behavior is determined by his or her behavioral intentions, which in turn are influenced by both personal attitudes and subjective norms. This study applies the Theory of Reasoned Action to the field of consumption and, combined with the characteristics of Millennials’ consumer behavior, constructs an extended Theory of Reasoned Action model (as shown in Figure 1). This model emphasizes that consumers are not only driven by internal attitudes in the decision-making process but also by external social norms.

This study aims to analyze the shift in consumer behavior among Millennials and further refine the influencing factors of consumer behavior intention. By integrating key factors in the consumption process, this study specifies the elements of the Theory of Reasoned Action model. In this model, consumer attitudes are represented by “consumer orientation” and “self-expression”; subjective norms are represented by “perceived economic pressures” and “perceived consumption risks”; and behavioral intentions are represented by “symbolic consumption perception”, “simulacrum consumption perception”, and “minimalist consumption consciousness”. Specifically, this study focuses on how consumer orientation, self-expression, perceived economic pressures, and perceived consumption risks affect symbolic consumption perception and simulacrum consumption perception and further analyzes the mechanism by which symbolic consumption perception and simulacrum consumption perception affect minimalist consumption consciousness. Ultimately, this study aims to reveal how these factors jointly influence minimalist consumption consciousness and an individual’s ultimate consumption behavior. On this basis, this paper will present in-depth exploration and analysis.

#### 2.1.2. Symbolic Consumption Perception and Simulacrum Consumption Perception

With the progression of science and technology and the evolution of consumer culture, the consumer behavior of Millennials is showing increasingly obvious symbolic and simulacra characteristics. Symbolic consumption and simulacrum consumption have become key perspectives for interpreting their consumption patterns. Scholars’ research on symbolic consumption focuses mainly on how consumer goods transcend their material functions and become carriers of social symbols and identity symbols. Among them, Veblen clearly proposed the concept of “conspicuous consumption” from an economic perspective; that is, the wealthy class displays their social status and power by consuming luxury goods. This laid the foundation for the study of symbolic consumption [11]. Sociologist Baudrillard further developed the theory of symbolic consumption. Baudrillard believed that in modern society, consumption is no longer simply a means of satisfying material needs but has become a tool for producing and consuming symbols, cultural values, and social identities [12]. In addition, Bourdieu, based on the theory of cultural capital, revealed that consumer behavior concerns not only an individual’s material needs but is also a profound social process that is a core component of social class, cultural identity, and identity construction [13]. Through the study of symbolic consumption, researchers have revealed the social, cultural, and psychological motivations behind consumer behavior and proposed that symbolic consumption is a behavioral pattern promoted by consumerist culture, in which consumers use the purchase of goods or services to convey personal social status, cultural values, and identity [14]. Consumption perception refers to the deeper psychological feelings and expressions of consumers about the products they consume [15]. As mentioned earlier, this study defines symbolic consumption perception as the totality of consumers’ psychological feelings and expressions about symbolic goods or services.

Building on the foundations of symbolic consumption, simulacrum consumption further explores the simulacrum value of commodities, emphasizing the blurring of the boundaries between the “real” and the “virtual” in modern consumption, as well as the profound impact of this transformation on consumer perceptions and behaviors. Baudrillard thoroughly explored the relationships between symbols, images, media, and consumption in modern society and first proposed the theory of “simulacra.” He believed that society had gradually entered an era of “hyperreality” in which consumption was not just about satisfying material needs, but also about consuming virtual, constructed meanings [16]. In the context of simulacrum consumption, consumers are not only the actual users of goods but also the recipients and re-producers of cultural symbols [17]. As Baudrillard predicted, in modern society, consumption is not only about material enjoyment but is also a way of constructing identity. The development of VR and AR further confirms the theory of simulacra. With the development of these emerging technologies, consumers are not just consuming goods but also experiences and meanings in the virtual world. Therefore, simulacrum consumption is a product of the collusion between mass media and consumerism, and its internal logic is to obscure reality and ultimately replace it [18]. As mentioned earlier, in conjunction with the study of simulacrum consumption, this study defines simulacrum consumption perception as the totality of consumers’ psychological feelings and expressions about simulacrum goods or services.

#### 2.1.3. Minimalist Consumption and Minimalist Consumption Consciousness

As modern society’s criticism of consumerism deepens, minimalist consumption is gradually emerging as an important trend in rethinking overconsumption. It advocates the concept of “less is more” and redefines the relationship between individuals and objects. Thoreau was one of the early representatives of the critique of materialism. He advocated a simple life, opposed the blind pursuit of material enjoyment, advocated returning to nature and the true self, and pursued inner peace and freedom, laying the theoretical foundation for minimalist consumption [19]. In the 1970s, Stigler and other scholars began to pay attention to the problem of consumerism in modern society, criticizing unnecessary consumption and the excessive penetration of advertising into people’s lives [20]. Since the 21st century began, minimalism has become a popular lifestyle, and minimalist consumption is its core practice. Minimalist consumption focuses on reducing unnecessary material possessions and excessive consumption, aiming to improve quality of life by not indulging in consumerism [21]. However, minimalist consumption is not just a simple behavioral choice, as it profoundly reflects a shift in consumer awareness. This shift is not only reflected in reducing one’s number of material possessions but also in re-examining the nature of consumption and deeply thinking about the quality of life. Minimalist consumption consciousness is at the heart of this shift in thinking, which encourages consumers to get rid of their over-reliance on material things, re-examine and evaluate the rationality of consumption decisions, and pay more attention to the satisfaction of intrinsic needs. Through this process, consumers not only reduce the burden on the environment and resources but also pursue a more minimalist and sustainable lifestyle. Consciousness is a psychological state involving self-awareness and perception of the external world, including an individual’s perception of and response to the environment, themselves, and their internal mental processes [22]. Based on this, combined with the research on minimalist consumption and consciousness, this study defines minimalist consumption consciousness as a socio-psychological phenomenon that focuses on minimalist consumption, reduces material needs, and improves quality of life.

In summary, symbolic consumption perception, simulacrum consumption perception, and minimalist consumption consciousness are intertwined in modern consumer culture. The consumer behavior of Millennials not only satisfies basic material needs but also profoundly reflects the construction of identity, social status, and cultural values. Under the dual challenges of consumerist culture and perceived economic pressures, Millennials have demonstrated a more diverse range of consumer attitudes. This study aims to explore the interplay between Millennials’ symbolic consumption perception, simulacrum consumption perception, and minimalist consumption consciousness and their impact on minimalist consumption behavior, as well as examining its long-term impact on personal well-being and social development.

### 2.2. Research Hypotheses

#### 2.2.1. Consumer Orientation, Perceived Economic Pressures, Self-Expression, and Perceived Consumption Risks

Consumer orientation refers to the values and attitudes that consumers display in their consumption behavior, which are mainly reflected in their preferences and pursuit of material enjoyment, enjoyment of life, brand identity, and other aspects [23]. Based on the analytical framework of the Theory of Reasoned Action, consumer orientation reflects consumers’ emotions and evaluations of their consumption behavior and is one of the important factors influencing their behavioral intentions. It not only includes consumers’ overall evaluation of their consumption behavior but also specifically refers to their emotional responses and psychological motivations. The consumer behavior of Millennials is no longer simply the satisfaction of functional needs but has become a key means of constructing social identity and cultural identity [24]. Symbolic consumption emphasizes the symbolic meaning of products in consumers’ choices, such as social status and identity, while simulacrum consumption focuses on consumers’ identification with the idealized, non-materialistic values represented by goods, such as the emotional resonance of idealized images conveyed in advertisements. When individuals’ consumer orientation is influenced by symbolic consumption, they tend to pay more attention to the symbolic value and social significance conveyed by these products when making choices. For example, Schroeder and Zwick’s research shows that consumer orientation affects individual consumption decisions, and that consumers’ symbolic consumption behavior is driven by cultural symbols and identity needs [25]. In addition, consumer orientation also affects consumers’ sensitivity to the value of product simulacra. Specifically, when consumers pursue virtual or immaterial values, they tend to pay more attention to the emotional value or simulacra meaning of the product. As Belk’s research shows, consumers in the digital age are increasingly inclined to associate their identity with virtual and immaterial assets. They are happy to buy virtual goods (such as digital art, virtual game items, or social media presence) and place more value on the simulacrum [26]. Therefore, the strengthening of consumer orientation has promoted the enhancement of Millennials’ symbolic consumption perception and simulacrum consumption perception.

Perceived economic pressures are individuals’ perceptions of current and future economic conditions. Specifically, perceived economic pressures mainly include two aspects: individuals’ perception of current economic difficulties and concerns about future economic conditions [27]. Within the analytical framework of the Theory of Reasoned Action, perceived economic pressures are an important factor affecting consumer intent. For example, Ajzen’s research shows that when consumers face an unstable economic environment, their behavior will rely more on rational assessment and actual needs rather than emotional consumer motives [28]. This means that consumers who perceive higher economic pressures will generally pay more attention to actual needs and necessities, and their consumer behavior will tend to be more rational. At the same time, it also suggests that perceived economic pressures may prompt individuals to become more sensitive to the symbolic and simulacrum value of consumer behavior. Specifically, when faced with limited economic resources, consumers will be more cautious in evaluating consumption decisions, especially for goods that lack direct practical value [29]. In the evaluation process, consumers not only pay attention to the actual utility of the goods but also increase their sensitivity to their emotional value and symbolic meaning. Therefore, perceived economic pressures make consumers more rational in making consumption decisions and increase their focus on the symbolic meaning and simulacrum value of goods.

Self-expression is the way in which people express their thoughts, preferences, and emotions [30]. For consumers, self-expression is the way in which they convey their personal identity, values, and emotions through objects, brands, behaviors, or other external symbols [31]. Millennials highly value personalization and uniqueness and are happy to express themselves in their lifestyles and consumer behavior. Therefore, in the consumer decision-making process, they often pay particular attention to the symbolic meaning and simulacrum value carried by goods and evaluate whether they can reflect their personal uniqueness, so as to avoid mass and homogeneous symbols or simulacrum goods. For example, they may avoid buying products with excessive commercialization or classic symbols (such as the iconic prints of luxury brands or classic IP images in blind boxes), because the symbolic meaning and simulacrum value of these goods are often diluted and homogenized in extensive consumption, thus losing their original uniqueness. As Kozinets and Handelman’s research further shows, with the increasing demand for self-expression, Millennials are increasingly inclined to choose consumer goods that reflect their individuality, uniqueness, and sense of social responsibility, rather than traditional mass symbolic goods or simulacra [32]. This choice reflects their sensitivity to the symbolic meaning and simulacrum value of consumer goods, as well as their high degree of concern about whether the goods can faithfully express their personal identity and social position. Therefore, the ability to express oneself not only affects Millennials’ consumer behavior but also enhances their symbolic and simulacrum consumption perception.

In consumer behavior research, perceived consumption risk is defined as the consumer’s expectation of subjective losses associated with possible options when making a purchase decision [33]. The Theory of Reasoned Action suggests that individuals will comprehensively consider the significance and consequences of their actions and the possible risks they may bring when making decisions. When Millennials perceive consumption risk in the consumer decision-making process, they hope to gain social recognition or avoid social exclusion through consumption, so they pay more attention to symbolic value. Research by scholars such as Lux shows that when individuals perceive a high consumption risk, they pay more attention to the symbolic value of goods and tend to choose those with obvious social symbolic significance to compensate for their concerns about possible future losses [34]. As perceived consumption risk increases, Millennials pay more attention to the simulacrum value of goods in the context of simulacrum consumption and gradually realize the illusory and transient nature of simulacrum value. Driven by perceived consumption risks, they will deeply realize that the psychological satisfaction brought by simulacrum consumption cannot solve practical problems but may instead bring more risks. As a result, they will more carefully evaluate the symbolic meaning and simulacrum value of goods to reduce unnecessary risks. To sum up, based on a rigorous literature review combined with an in-depth discussion of the mechanisms of consumer orientation, perceived economic pressures, self-expression, and perceived consumption risks on symbolic consumption perception and simulacrum consumption perception, this paper proposes the following hypotheses:

**H1:**  *Consumer orientation has a positive impact on symbolic consumption perception and simulacrum consumption perception*.

**H2:**  *Perceived economic stress positively affects symbolic consumption perception and simulacrum consumption perception*.

**H3:**  *Self-expression positively affects symbolic consumption perception and simulacrum consumption perception*.

**H4:**  *Perceived consumption risk positively affects symbolic consumption perception and simulacrum consumption perception*.

#### 2.2.2. The Influence of Symbolic Consumption Perception and Simulacrum Consumption Perception on Minimalist Consumption Consciousness

According to the Theory of Reasoned Action, consumers take into account various pieces of information when making decisions in order to make rational choices. This is also true of Millennials, who weigh the significance and consequences of their decisions during the consumption process. For Millennials, the perception of symbolic consumption reinforces their focus on the symbolic value of goods, enabling them to distinguish between the use value and symbolic value of goods. As their perception of symbolic consumption increases, Millennials gradually realize that the essence of symbolic consumption is the pursuit of status symbols and social identity, and that this form of consumption often lacks practical functionality and is of low cost performance, unable to bring sufficient use value or satisfaction. As Batra’s research shows, the stronger the perception of symbolic consumption, the more consumers are aware of the irrationality of consumption, and thus, they spontaneously pursue more simple and meaningful approaches to consumption [35]. Therefore, Millennials will begin to reflect on the necessity of symbolic consumption and rationally evaluate consumption decisions to see if they are made to satisfy real needs or to pursue social approval or status symbols. Therefore, as the perception of symbolic consumption increases, Millennials will pay more attention to the essence of consumer behavior, focus on the actual value of goods, and tend to have a rational and minimalist consumption consciousness.

Similarly, simulacrum consumption will increase Millennials’ attention to the emotional value and simulacrum meaning of goods. As their perception of simulacrum consumption deepens, Millennials’ ability to distinguish between the use value and simulacrum value of goods is enhanced. For example, Richins’ research shows that when making consumption decisions, individuals often intertwine emotional needs with material needs and may even place the emotional value of an object above its material value [36]. However, when Millennials realize that the simulacrum value of a product (such as an idealized lifestyle) offers limited satisfaction of their intrinsic needs, they will reflect on the true motivation for this consumer behavior and be vigilant against non-essential emotional gratification. As Loewenstein and O’Donoghue’s research points out, individuals may adjust their consumer behavior over time through a process of reflection and self-awareness. Once they realize through experience or reflection that emotionally driven consumer decisions do not bring lasting practical benefits, they may reduce emotionally driven consumption and pay more attention to rational analysis [37]. As simulacrum consumption perception deepens, consumers tend to reduce their reliance on overconsumption and instead pay more attention to the actual use value and long-term significance of goods, thus promoting the formation of minimalist consumption concepts. As Schor and White’s research shows, consumers’ increased sensitivity to simulacrum consumption can promote their shift to a more rational and minimalist consumption model [38]. Therefore, simulacrum consumption perception helps stimulate consumers’ identification with minimalist consumption concepts and promote the formation and strengthening of minimalist consumption consciousness. Based on this, the following hypotheses are proposed:

**H5:**  *Symbolic consumption perception has a positive impact on minimalist consumption consciousness*.

**H6:**  *Simulacrum consumption perception has a positive impact on minimalist consumption consciousness*.

#### 2.2.3. The Impact of Minimalist Consumption Consciousness on Consumer Behavior and the Moderating Effect of Upward Social Comparison

Minimalist consumption consciousness is a set of values that emphasizes enhancing an individual’s spiritual life and sense of well-being by reducing material needs. Its core concept is to focus on the quality, sustainability, and long-term use value of objects, rather than short-term consumer satisfaction. According to the Theory of Reasoned Action, behavioral intention is a key factor in achieving actual behavior. The stronger the behavioral intention, the more likely an individual is to turn it into action. As Cherrier’s research shows, minimalist consumption consciousness can encourage consumers to pay more attention to the durability and practicality of goods, reduce unnecessary purchases, and prompt them to adopt a simplified lifestyle and more-sustainable consumption patterns [39]. Specifically, when an individual’s behavioral intention is influenced by minimalist consumption consciousness, they are likely to make fewer impulse purchases and tend to choose products that align with their values. As White et al. have shown, minimalist consumption consciousness often prompts individuals to re-examine their own consumption motives. In particular, when they begin to adopt a higher-level way of thinking, this consumption behavior will develop in a more rational and simplified direction [40]. For Millennials, minimalist consumption consciousness has gradually shifted their examination of consumer behavior towards rational decision making, with many actively avoiding over-consumption and choosing goods that can be used for a long time and have high utility value [41]. Therefore, the rise of minimalist consumption consciousness will prompt Millennials to make consumer choices that align with their needs and values and will positively promote the realization of minimalist consumption behavior.

**Figure 2:**
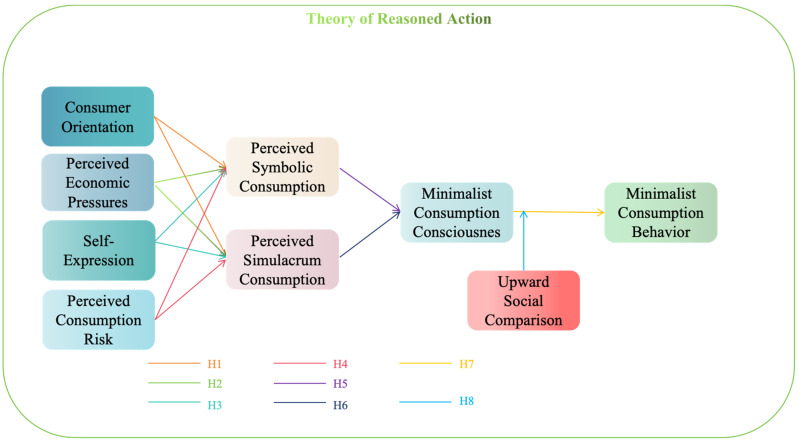
Proposed model diagram.

In the 1950s, Festinger proposed the theory of social comparison, which states that social comparison is a state in which humans evaluate their own opinions and abilities in comparison to others, which is an important function for establishing self-identity [42]. For Millennials, the rise of minimalist consumption consciousness has prompted them to tend towards minimalism in their consumer behavior. However, during this transition process, upward social comparison acts as an important moderating factor that regulates this behavior. For example, Kruglanski and Mayseless’s research shows that people are naturally inclined to compare themselves with others to assess their status and abilities [43]. Specifically, Millennials will inevitably engage in upward social comparison in their daily lives. Influenced by “face” and “family honor,” Millennials are compared with their peers even as children. As Liu et al. point out, the expectations of parents for their children in Chinese families drive upward social comparison. Parents often expect their children to surpass their peers and become the pride of the family, and this expectation prompts children to compare themselves with “other people’s children,” especially in terms of academic achievement and career progression [44]. Richins’s research further shows that while the popularity of social media exposes young people to more “ideal lives,” it also makes them engage in upward social comparison more frequently, which in turn affects their consumption decisions [45]. When Millennials realize the gap between themselves and others in terms of wealth, family, career, etc., negative emotions can exacerbate feelings of dissatisfaction, leading to irrational consumption decisions. For example, driven by an idealized image, they may develop a purchase motive in order to bridge the gap with the idealized image, trying to enhance their social status through material consumption and avoid being regarded as “unsuccessful” or “outdated.” For example, the results of Liu et al.’s research show that upward social comparison can lead to more impulse buying behavior [46]. In summary, upward social comparison often weakens the impact of minimalist consumption consciousness on consumer behavior, causing the consumer behavior of Millennials to deviate from the minimalist concept. Based on this, the following hypothesis is proposed (the specific proposed model in this paper is shown in Figure 2):

**H7:**  *Minimalist consumption consciousness has a positive influence on minimalist consumption behavior*.

**H8:**  *Upward social comparison negatively regulates the impact of minimalist consumption consciousness on minimalist consumption behavior*.

## 3. Research Design

### 3.1. Subjects and Questionnaire Distribution

This study focuses on the changing consumer behavior of Millennials. Due to the limitations of obtaining offline research samples, this study used the Credamo survey platform to generate a questionnaire link and invited Millennials to participate in filling it out via email, social media, and other methods. The questionnaire was further promoted through “snowballing” to ensure that the sample covered a wide range of diversity. The questionnaire was anonymous and strictly followed the Declaration of Helsinki to ensure that participants volunteered to participate and could stop filling out the questionnaire at any stage.

Before the official launch, the research team first tested a small offline sample of 26 people in Nanjing, China, to assess the logic, clarity, and applicability of the questionnaire to the changing consumption behavior of Millennials. Based on the feedback from the test, the questionnaire was adjusted accordingly to ensure that it accurately reflected the research objectives. A total of 600 questionnaires were distributed for this study. This figure represents the total number of questionnaires sent out via email, social media, and the Credamo platform. Of these, 572 questionnaires were actually returned, while some of the questionnaires sent out were not filled in or were not submitted for other reasons. Of the 572 questionnaires received, invalid questionnaires (such as those with short answers, incomplete entries, duplicate submissions, logical inconsistencies, etc.) were removed after cleaning, and 554 valid questionnaires were retained, with an effective rate of 96.85%, ensuring the quality of the data.

The sample of this study covers the multi-dimensional backgrounds of Millennials, such as gender, age, and region of permanent residence. In addition to the usual basic information, this article innovatively considers participants’ the field of specialization after graduation. This is because there are differences in the educational background and knowledge structure between liberal arts and science graduates, which may have a potential impact on their consumption behavior. Therefore, ensuring that the sample includes individuals from both liberal arts and science backgrounds helps to obtain more representative data, avoid bias in the research results due to the emphasis on a particular professional category, and make the sample more representative and balanced (see Table 1 for details).

### 3.2. Variable Measurement

This study draws on the measurement of consumption orientation, perceived economic pressure, self-expression, perceived consumption risk, and upward social comparison from the established scales of Webster et al. [47], Roberts et al. [48] and Webb et al. [49]; Cohen et al. [50] and Wadsworth et al. [51]; Robbina [52]; Mitchell et al. [53] and Peter et al. [54]; and Gibbons et al. [55], respectively, with simple modifications based on real-life situations.

Current measurements of minimalism mainly rely on the Materialism Values Scale (MVS) developed by Richins [56] and others. This study eliminates irrelevant dimensions and draws on the scales of Chaudhuri et al. [57] and Iyer et al. [58] to form a 6-item scale of minimalist consumption awareness and minimalist consumption behavior that is consistent with the Chinese context based on a sample of Millennials. For example, included in the scale are statements like “I will not be attracted by marketing or promotions to buy things I don’t need”.

Since previous research has rarely studied the specific measurement of symbolic consumption perception and simulacrum consumption perception, this study conceptually draws on the connotative explanations of Schouten et al. [59], Langley et al. [60], and Hubbs et al. [61] for the measurement of the two metrics. This study proposes that under the influence of online information dissemination, the consumption motives of Millennials have gradually changed, and their consumption behaviors and patterns have also shown changes such as “symbolization”. The counterproductive effect of today’s trendy consumer goods on Millennials’ consumption behaviors, attitudes, and even values is the final stage of simulacra: the contemporary representation of reality. With this understanding, combined with the current real-life consumption habits and psychology of Chinese Millennials, a 6-item questionnaire scale was designed to target symbolic consumption perception and simulacrum consumption perception, including such statements as “I think some of the current consumption hotspots are exaggerating the symbolic meaning of goods or services” and “I don’t think the new way of consuming has defined us or constructed our self-identity.” Compared to a 7-point or 10-point scale, a 5-point Likert scale has better balance and is neither overly complicated nor loses its sensitivity to capturing the attitudes of the subjects. It also shows improved questionnaire completion rate; studies have shown that long or complex scales tend to reduce the willingness of subjects to complete them, while the 5-point Likert scale can improve the accuracy of responses and the completion rate of the questionnaire by reducing cognitive load [62,63]. Therefore, the Likert 5-point scale was used in this study, with scores from 1 to 5 representing “strongly disagree” to “strongly agree”.

## 4. Research Results

### 4.1. Reliability and Convergent Validity

The data from the pre-survey questionnaire were tested using item analysis, and the questionnaire data were tested for item–total score correlation using SPSS 26.0 and for reliability using Cronbach’s Alpha (CA). It is generally considered that the Cronbach’s Alpha coefficient must be greater than 0.7 for variables to have good reliability. Convergent validity refers to the degree of similarity between the measurement results when the same characteristic is measured using different measurement methods; that is, different measurement methods should converge in the measurement of the same characteristic. Convergent validity is indicated when the factor load of each measurement question is greater than 0.7, the composite reliability of each dimension is greater than 0.7, and the average variance extracted of each dimension is greater than 0.5.

As can be seen in Table 2, the Cronbach’s Alpha value of each variable in this study is greater than 0.7, indicating that each variable has good reliability. In addition, the CR value is greater than 0.7, and the factor load of each measurement question is greater than 0.7, indicating good indicator reliability. The AVE value is greater than 0.5, indicating that each variable has good convergent validity.

### 4.2. Discriminant Validity

The discriminant validity of the measurement model can be tested using the Fomell–Larcker criterion and the HTMT method. The Fomell–Larcker criterion tests discriminant validity by observing the magnitude of the square root of the average extracted variance (AVE) and the correlation coefficient between the latent variables. If the square root of the AVE value is greater than the correlation coefficient between each potential variable (i.e., the diagonal values are greater than the off-diagonal values), this indicates that the measurement model has good discriminant validity between each latent variable, and if the HTMT value is less than 0.9, this indicates discriminant validity. Table 3 and Table 4 show that the square root of the AVE for each variable is greater than the correlation coefficient between each variable, and that the HTMT value for each variable is also less than 0.9, indicating that the variables in this study have discriminant validity.

### 4.3. Fit Test

In order to further verify whether the questionnaire is consistent with the expected theory, the SmartPLS 4.0 software was used to analyze the questionnaire to see if a measurement item has a significant load on the corresponding factor and no significant load on irrelevant factors. The analysis results show that the SRMR is 0.039, which is less than 0.05, indicating an ideal fit; the NFI is 0.859, which meets the 0.8 standard, indicating a good fit. Therefore, it can be concluded that the model has a good fit.

### 4.4. Common Method Deviation

The Harman one-factor test was used to test for common method deviation. Generally, as long as the percentage of variance explained by the first common factor is less than 40%, it can be considered that there is no serious common method deviation. It was found that the first factor in this study explained 38.444% of the variance, which is less than the critical value of 40%. This indicates that there is no significant common method deviation in this study.

### 4.5. Multiple Co-Linearity Test

High multiple collinearity will increase parameter estimates and reduce the accuracy of the proposed model. To determine whether this indicator meets the requirements of the study, the Variance Inflation Factor (VIF) is usually used for testing. As can be seen from Table 5, the VIF values of the variables in this study are all less than five, indicating that there is no multiple collinearity between the independent variables.

### 4.6. Structural Equation Model Estimation

**Figure 3:**
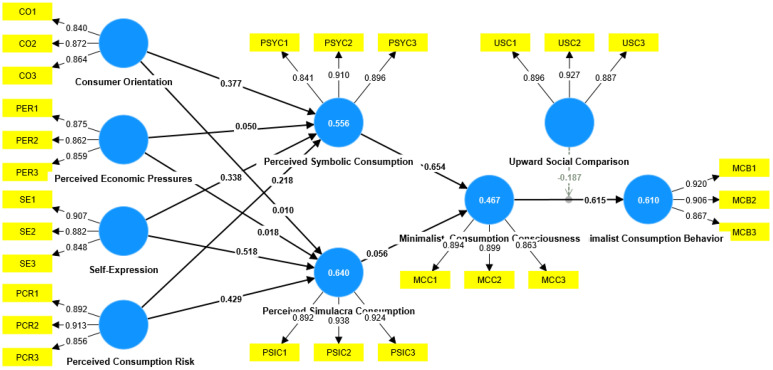
Corrected PLS−SEM results plot.

This study used Smart PLS 4.0 statistical analysis software to test the collected data using partial least squares structural equation modeling (PLS-SEM). Partial least squares is a multivariate statistical data analysis method that finds the best functional match for a set of data by minimizing the square of the error. It can model the regression of multiple dependent variables on multiple independent variables. In PLS analysis, the test of the structural model includes the estimation of path coefficients and the values of R^2^ and Q^2^. Path coefficients reflect the direction and degree of influence between latent variables. The R^2^ value reflects the degree to which endogenous latent variables in the structural model can be explained by exogenous latent variables and also reflects the explanatory power of the model. Q^2^ is the correlation between predicted manifest variables. In accordance with the theoretical model constructed in this chapter, in order to verify the model and hypotheses proposed in this study, we used the visual Smart PLS 4.0 to perform PLS analysis and the Bootstrapping sampling method to calculate the significance of the path coefficients in the constructed model (as shown in Figure 3). As can be seen in Table 6, the R^2^ values of simulacrum consumption perception, minimalist consumption awareness, minimalist consumption behaviors, and symbolic consumption perception are 0.640, 0.467, 0.610, and 0.556, respectively, indicating moderate explanatory power; the Q^2^ values are 0.535, 0.364, 0.484, and 0.427, respectively, all of which are greater than zero, indicating positive predictive power.

### 4.7. Path Coefficient

**Figure 4:**
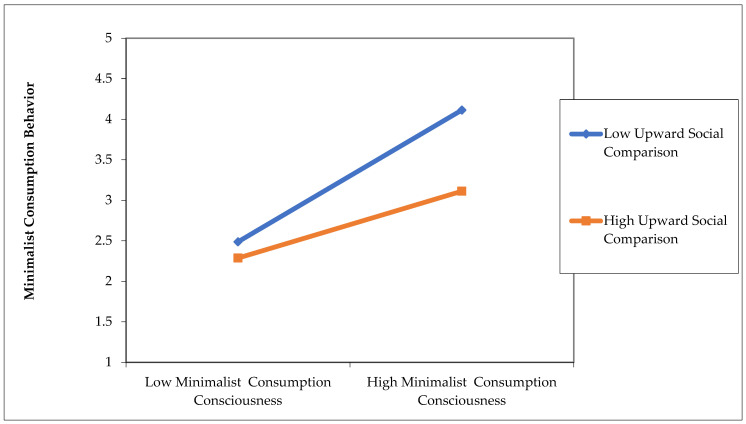
Regulatory effect diagram.

As can be seen in Table 7, consumption orientation via symbolic consumption perception (β = 0.377, *p* < 0.05) has a significant positive impact, so the related hypothesis is established; perceived economic pressure via symbolic consumption perception (β = 0.05, *p* > 0.05) does not have a significant positive impact, so the related hypothesis is not established; self-expression via symbolic consumption perception (β = 0.338, *p* < 0.05 has a significant positive impact, so the related hypothesis is established; perceived consumption risk via symbolic consumption perception (β = 0.218, *p* < 0.05) has a significant positive impact, so the related hypothesis is established; consumption orientation via simulacrum consumption perception (β = 0.010, *p* > 0.05) does not have a significant positive impact, so the related hypothesis is not established; perceived economic pressure via simulacrum consumption perception (β = 0.018, *p* > 0.05) does not have a significant positive impact, so the related hypothesis does not hold; self-expression via simulacrum consumption perception (β = 0.518, *p* < 0.05) has a significant positive impact, so the related hypothesis is established; perceived consumption risk via simulacrum consumption perception (β = 0.429, *p* < 0.05) has a significant positive impact, so the related hypothesis is established; symbolic consumption perception via minimalist consumption awareness (β = 0.654, *p* < 0.05) has a significant positive impact, so the related hypothesis is established; simulacrum consumption perception via minimalist consumption awareness (β = 0.056, *p* > 0.05) does not have a significant positive impact, so the related hypothesis is not established; minimalist consumption awareness via minimalist consumption behavior (β = 0.615, *p* < 0.05) has a significant positive impact, so the related hypothesis is established;

Upward social comparison of minimalist consumption awareness via minimalist consumption behavior (β = −0.187, *p* < 0.05) has a significant negative impact, indicating that upward social comparison has a negative moderating effect between minimalist consumption awareness and minimalist consumption behavior. The hypothesis is established, and the moderating effect is shown in Figure 4.

In this study, the independent variables include consumption orientation, perceived economic pressure, self-expression, perceived consumption risk, symbolic consumption perception, simulacrum consumption perception, and upward social comparison. These independent variables directly affect the dependent variables, which are minimalist consumption awareness and minimalist consumption behavior. Among them, independent variables such as consumption orientation, self-expression, and perceived consumption risk affect minimalist consumption awareness by changing individuals’ perceptions and attitudes towards consumption, which in turn further affects minimalist consumption behavior. In addition, upward social comparison, as a moderating variable, plays a moderating role between minimalist consumption awareness and minimalist consumption behavior, weakening the positive relationship between the two. In order to intuitively determine whether the hypotheses are valid, this paper presents a hypothesis result table to visually determine whether the original hypotheses are valid (see Table 8 for details).

## 5. Discussion

### 5.1. The Original Hypothesis That Some Paths Are Not Significant

By analyzing the main factors influencing the changing consumer behavior of Millennials, some paths were found to not be significant. One of the most surprising findings was that perceived economic pressures did not significantly affect either symbolic consumption or simulacrum consumption. One possible explanation is that perceived economic pressures did not significantly reduce Millennials’ demand for symbolic consumption and simulacrum consumption. This can be explained by Bauer’s research, which shows that in situations of high economic pressure, consumers often purchase luxury goods to enhance their social status [64]. This conclusion is further supported by the research of Hill et al., who found that even during economic downturns, women’s spending on beauty products did not decrease, while men’s desire to buy luxury goods may increase during economic downturns [65,66]. This shows that even in an environment with high economic pressure, Millennials still pursue goods with high social symbolic value or simulacrum value. In addition, we found that China’s unique cultural background may have played an important role. Millennials often receive financial support from their parents or elders (such as red envelopes and gifts) [67], which to some extent alleviates the perceived economic pressures they face and weakens the impact of economic pressures on consumption perception. Therefore, perceived economic pressures did not significantly affect Millennials’ symbolic consumption or simulacrum consumption perceptions.

As well as perceived economic pressures, the influence of consumer orientation on Millennials’ simulacrum consumption perception was also found to be insignificant. This may be attributed to the fact that the simulacrum value of goods is relatively hidden and not easily discovered by Millennials. Specifically, the value of many simulacrum goods is often virtual and obscure in nature, lacking specific functional features, which makes Millennials’ perception of simulacrum value more ambiguous. Therefore, in the context of simulacrum consumption, Millennials have difficulty discerning the simulacrum value and use value of goods, which in turn weakens the impact of consumer orientation on simulacrum consumption perception.

Finally, simulacrum consumption perception has no significant effect on minimalist consumption consciousness, which may be due to the virtual nature of simulacrum consumption. For example, forms of consumption favored by Millennials, such as online game items or digital consumption, do not involve the occupation of physical space and therefore lack a direct connection to the need for material simplicity, making it difficult for simulacrum consumption perception to have a direct impact on minimalist consumption. In addition, for Millennials, simulacrum consumption greatly satisfies their need for emotional value. For example, Zhang’s research shows that simulacrum consumption provides Millennials with a way to relax, have fun, or escape stress, helping them relieve tension related to their studies, work, and lives [68]. However, after Millennials perceive a higher emotional value or simulacrum significance in a product, it is not easy to trigger their self-reflection or restraint regarding material consumption. Therefore, simulacrum consumption perception is not an effective way to stimulate Millennials’ identification with the minimalist consumption concept, and its impact on minimalist consumption consciousness is relatively limited.

### 5.2. Analysis of the Influencing Factors of Symbolic and Simulacrum Consumption Perception

By analyzing the influencing factors of Millennials’ symbolic consumption perception and simulacrum consumption perception, we have come to the following conclusions: First, consumer orientation has a significant positive impact on symbolic consumption perception. This is because Millennials are increasingly concerned about the symbolic value of goods. For example, Dittmar’s research results show that individuals consume symbols as a way to shape their self-identity and pursue happiness. Symbolic consumption not only reflects their social status and personal identity but is also closely linked to their pursuit of a “perfect” life and body [69]. Millennials, who see symbolic goods as a means of displaying their identity and social belonging, are particularly sensitive to the symbolic value of goods. Consumer orientation, by strengthening the symbolic value inherent in goods, guides Millennials to pay more attention to the symbolic attributes of goods in the consumption process, thereby enhancing their symbolic consumption perception.

Second, self-expression has a significant positive impact on both symbolic consumption perception and simulacrum consumption perception. This phenomenon reflects Millennials’ strong desire for personalized expression and identity construction. Specifically, as their self-expression ability improves, Millennials show a higher demand for personalized goods and are more inclined to purchase unique symbolic or simulacrum goods. Therefore, they will further deepen their perception of symbolic consumption and simulacrum consumption to evaluate whether limited-edition products can show their unique identity and taste [70]. As mentioned earlier, driven by self-expression, Millennials have a keener perception of symbolic consumption and simulacrum consumption. However, Schau and Gilly pointed out that the act of self-expression drives consumers to indulge in symbolic consumption, but this behavior does not directly enhance the perception of the symbolic value of goods [71]. After analyzing their results, we found that Schau and Gilly’s research subjects were not Millennials, and the research context was also different to that of this study. They mainly explored the relationship between self-expression and symbolic consumption based on personal web spaces.

Finally, perceived consumption risk had a significant positive impact on symbolic consumption perception and simulacrum consumption perception. This finding is consistent with existing research and further verifies that consumers generally tend to avoid risk in the consumer decision-making process, while their willingness to buy may increase with a reduction in risk [72]. The pursuit of pleasure and risk aversion are basic human psychological responses. Especially in high-risk consumption situations, Millennials are more inclined to adopt a rational assessment approach and comprehensively consider the multiple values of goods. This assessment is not limited to the actual use value of the product but also includes its symbolic meaning and simulacrum value, which makes them more cautious in decision making. During the research phase, some respondents admitted that after perceiving a higher consumption risk, they become more rational and keenly perceive the symbolic or emotional value of the product, so as to make a more rational consumption choice.

### 5.3. Symbolic and Simulacrum Consumption Perception and Minimalist Consumption Awareness

When exploring the influence of Millennials’ symbolic consumption perception and simulacrum consumption perception on minimalist consumption consciousness, we found that symbolic consumption perception has a significant positive effect on minimalist consumption consciousness. As the phenomenon of “symbolization” becomes increasingly prominent in daily life, Millennials have gradually come to realize that overemphasizing the “symbolic value” of goods while ignoring their actual use value is likely to lead to irrational and even unhealthy consumer attitudes, which in turn places a burden on their lives. At the same time, minimalism, as an increasingly popular consumer trend, has prompted these young people, who have a strong perception of symbolic consumption, to reflect on their own consumer behavior. They have begun to shift towards minimalist consumption patterns, forming and strengthening minimalist consumption consciousness, in an attempt to avoid the problems of overconsumption and materialism. However, Klein and Dawar’s research shows that symbolic consumption has a powerful appeal, and consumers often pay more attention to the identity, status, personal brand, or lifestyle symbolism represented by the product than to the actual functionality of the product. Even if, after rational analysis, the cost performance of the product is not outstanding, consumers may still be immersed in the symbolic consumption experience and not switch to a minimalist consumption model [73]. Their research focuses on how corporate social responsibility affects consumer behavior and indirectly explores the relationship between symbolic consumption and the symbolic value and practical utility of products. Therefore, there are some differences between their research results and this study, which mainly stem from the different frameworks used—this study focuses more on the direct impact of symbolic consumption perception on minimalist consumption consciousness, while Klein and Dawar mainly explore consumer behavior in the context of corporate social responsibility.

### 5.4. Minimalist Consumption Consciousness and Minimalist Consumption Behavior

Minimalist consumption consciousness has a positive impact on minimalist consumption behavior. In recent years, the driving force of minimalist consumption consciousness on minimalist consumption behavior has become increasingly significant, especially among Millennials. This generation is increasingly inclined to choose high-quality goods with long-term value and functionality and prefers to focus on the durability, functionality, and practicality of goods, rather than pursuing quantitative satisfaction. In addition, Millennials’ concern for social responsibility, such as environmental protection and sustainable development, has further promoted their tendency to reduce unnecessary consumption and choose brands and products that are consistent with their values. As Bardhi and Eckhardt’s research shows, when consumers have a minimalist consumption consciousness, they tend to seek consumption methods that reduce personal resource occupation and material accumulation. The sharing economy (such as car sharing) allows consumers to enjoy the convenience of goods without owning them [74]. Therefore, consumers’ positive promotion of their behavior by giving up the possession of material excess and choosing to use them on demand reflects the minimalist consumption consciousness.

### 5.5. The Moderating Effect of Upward Social Comparison

Upward social comparison is a key moderating factor in the relationship between minimalist consumption consciousness and behavior, and the reasons for its negative moderating effect can be attributed to three factors. First, Millennials are susceptible to social pressure and expectations, especially when engaging in upward social comparisons with people who have higher social status, more wealth, or greater achievement. This social pressure often prompts them to blindly pursue higher consumption levels to conform to mainstream social standards, thereby weakening their minimalist consumption consciousness. In addition, the development of social media platforms has exacerbated this phenomenon. For example, Schmuck et al. showed that behaviors dedicated to creating a “perfect persona” may reduce Millennials’ self-esteem and happiness, which in turn intensifies their desire to consume [75]. Second, upward social comparison is likely to shake Millennials’ self-identity. When they see that others have more resources, they may feel discontent and anxious, which in turn reduces their minimalist consumption consciousness and leads them to pursue material enjoyment. “Peer pressure” in social networks often translates to impulsive consumption, which in turn prompts young people to make irrational consumption decisions. Finally, upward social comparison is more likely to stimulate envy and the psychology of comparison, driving Millennials to imitate those who are more successful and show their social status through increased consumption. This psychological drive causes them to deviate from the original intention of minimalist consumption. To sum up, upward social comparison negatively moderates the impact of minimalist consumption consciousness on minimalist consumption behavior through social pressure, self-identity instability, and the psychology of comparison.

## 6. Conclusions

This paper analyzes the causes of changes in the consumption habits of Chinese Millennials using a questionnaire survey and the PLS-SEM method. The results show that self-expression and perceived consumption risk have a significant positive impact on symbolic consumption perception and simulacrum consumption perception, indicating that Millennials pay more attention to the symbolic meaning and simulacrum value of goods when pursuing individuality and avoiding risks. Consumption orientation significantly and positively affects symbolic consumption perception but has no significant effect on simulacrum consumption perception, indicating that Millennials are more likely to recognize the symbolic value of goods and are less sensitive to the implied simulacrum value. At the same time, perceived economic pressure has no significant effect on either type of consumption perception, indicating that economic pressure does not directly change their consumption attitudes. In addition, symbolic consumption perception significantly enhances minimalist consumption awareness, indicating that while Millennials value symbolic consumption, they are gradually embracing a minimalist lifestyle. Upward social comparison has a negative moderating effect on the relationship between minimalist consumption awareness and behavior; that is, when comparing themselves with those who are better off, the impact of minimalist awareness on Millennials’ actual behavior will be weakened. This finding provides empirical support for the theory of social comparison and reveals the complexity and diversity of Millennials’ consumption behavior against the backdrop of downward economic pressure.

## 7. Implications

This study makes important theoretical contributions to the consumption of symbols and simulacra, minimalist consumption, and consumer psychology. First, this paper introduces the dual perception of symbolic consumption and simulacrum consumption into the analytical framework of minimalist consumption behavior, expanding the theoretical perspective of the field of consumer behavior research and revealing how Millennials complete self-representation through symbolic and simulacrum consumption in a complex economic environment. This study extends the applicability of symbolic consumption and simulacrum consumption in times of economic downturn and provides a theoretical basis for future consumer behavior research. Second, this study is the first to verify the negative moderating effect of upward social comparison on minimalist consumption behavior, demonstrating how minimalist consumption consciousness is weakened under social comparison pressure, which leads to deviations in consumption behavior. This provides a new explanatory mechanism for understanding consumption behavior in periods of economic downturn and opens up new directions for future research on social influence factors in minimalist consumption research.

In addition to the above theoretical significance, this study also has rich management significance. First, this study provides a reference for companies to gain insight into minimalist consumption trends and develop scientific marketing strategies. Especially against the backdrop of increasing downward pressure on the global economy, this study reveals the complex psychological mechanisms behind the minimalist consumption behavior of Millennials, helping companies analyze the needs of Millennials and providing new theoretical ideas for stimulating the global consumer market. Companies can appropriately leverage the social comparison effect to enhance the market positioning of symbolic goods and enhance consumers’ sense of identity. At the same time, through “anti-luxury consumption” marketing methods, they can encourage a minimalist lifestyle and adapt to the changing consumption concepts of Millennials. Second, this study provides a reference for governments and social institutions to formulate policies. Research shows that economic pressure significantly affects consumer behavior, which suggests that policymakers should alleviate the economic pressure on Millennials by improving social security and employment stability, promote healthy consumption, and stimulate domestic demand. In addition, the government can promote the concepts of green, low-carbon consumption and minimalist living to promote sustainable social development.

## 8. Limitations

Due to limitations regarding research costs and the accessibility of the target group, this study used a “snowball” method to promote the questionnaire and ultimately collected 554 valid questionnaires. Although this sample provides useful insights into the changing consumer behavior of Millennials, there are still some limitations to the study. First, the survey sample was mainly concentrated in the eastern, central, and western regions of China and did not cover other ethnic, national, or international groups. Therefore, although the research results are somewhat representative, they are somewhat limited in their general applicability to the changing minimalist consumption behavior of Millennials in China and globally. Second, Chinese culture is more likely to influence the results and expected behavior of the subject group, as some research results show that in Western countries, young people are more susceptible to materialism. Therefore, the global representativeness of the research results needs to be further verified. Since this study is based on a questionnaire survey and cross-sectional data, it may be affected by social desirability bias and recall bias, which may affect the accuracy and reliability of the results. Finally, this study mainly used quantitative analysis methods and failed to explore the deep-seated motivations and psychological mechanisms behind the consumer behavior of Millennials. Future research should not only contribute to a more comprehensive understanding of the consumer behavior of Millennials around the world but also provide a more accurate reference basis for policymakers and businesses’ marketing practices. First, the sample size can be expanded to include Millennials from different cultural backgrounds and regions, thereby improving the representativeness of the data. Cross-cultural comparative research can be used to more fully understand the differences in consumer behavior among Millennials around the world. Second, a longitudinal study can be designed using a combination of quantitative and qualitative research methods, and panel data can be used to conduct an in-depth analysis of the internal mechanisms of minimalist consumption behavior in order to reveal its evolution process and driving factors. Finally, in-depth interviews and case studies can be used to further explore the psychological motivations behind the consumer behavior of Millennials.

## Figures and Tables

**Table 1:** Descriptive statistical analysis table.

Causality	Form	Number of People	Percentage
Sex	Male	267	48.2
	Female	287	51.8
Birth Year	1980–1983	13	2.3
	1984–1986	191	34.5
	1987–1990	153	27.6
	1991–1994	108	19.5
	1995–1997	82	14.8
	1998–1999	7	1.3
Eternal Residence	East	292	52.7
	West	262	47.3
Major field after graduation	Science major	296	53.4
	Liberal arts major	258	46.6
Account Type	Municipalities	214	38.6
	Countryside	340	61.4
Marital Status	Married	431	77.8
	Unmarried or divorced	123	22.2

**Table 2:** Table of reliability and convergent validity.

Variable	Item	Loading	Cronbach’s Alpha	CR	AVE
Consumer Orientation (CO)	CO1	0.840	0.822	0.894	0.737
	CO2	0.872			
	CO3	0.864			
Minimalist Consumption Behavior (MCB)	MCB1	0.920	0.880	0.926	0.806
	MCB2	0.906			
	MCB3	0.867			
Minimalist Consumption Consciousness (MCC)	MCC1	0.894	0.862	0.916	0.784
	MCC2	0.899			
	MCC3	0.863			
Perceived Consumption Risk (PCR)	PCR1	0.892	0.865	0.918	0.788
	PCR2	0.913			
	PCR3	0.856			
Perceived Economic Pressure (PEP)	PER1	0.875	0.832	0.899	0.749
	PER2	0.862			
	PER3	0.859			
Perceived Simulacrum Consumption (PSC)	PSIC1	0.892	0.907	0.942	0.843
	PSIC2	0.938			
	PSIC3	0.924			
Perceived Symbolic Consumption (PSC)	PSYC1	0.841	0.858	0.914	0.779
	PSYC2	0.910			
	PSYC3	0.896			
Self-Expression (SE)	SE1	0.907	0.854	0.911	0.774
	SE2	0.882			
	SE3	0.848			
Upward Social Comparison (USC)	USC1	0.896	0.888	0.930	0.816
	USC2	0.927			
	USC3	0.887			

**Table 3:** Fornell–Larcker criterion.

	USC	PCR	PEP	PSIC	MCC	MCB	CO	PSYC	SE
USC	0.904								
PCR	−0.301	0.888							
PEP	−0.440	0.400	0.865						
PSIC	−0.393	0.636	0.383	0.918					
MCC	−0.400	0.241	0.242	0.390	0.886				
MCB	−0.556	0.135	0.205	0.289	0.696	0.898			
CO	−0.488	0.412	0.384	0.378	0.402	0.330	0.859		
PSYC	−0.425	0.520	0.405	0.511	0.682	0.451	0.607	0.883	
SE	−0.380	0.377	0.366	0.690	0.363	0.288	0.357	0.573	0.880

**Table 4:** HTMT.

	USC	PCR	PEP	PSIC	MCC	MCB	CO	PSYC	SE
USC									
PCR	0.345								
PEP	0.512	0.471							
PSIC	0.438	0.717	0.441						
MCC	0.455	0.279	0.286	0.440					
MCB	0.627	0.155	0.238	0.323	0.798				
CO	0.571	0.487	0.462	0.437	0.478	0.387			
PSYC	0.488	0.603	0.480	0.579	0.792	0.517	0.723		
SE	0.435	0.436	0.432	0.783	0.423	0.331	0.423	0.668	

**Table 5:** VIF Values.

	PSIC	MCC	MCB	PSYC
USC			1.265	
PCR	1.373			1.373
PEP	1.334			1.334
PSIC		1.354		
MCC			1.270	
MCB				
CO	1.339			1.339
PSYC		1.354		
SE	1.290			1.290
USC × MCC			1.092	

**Table 6:** R^2^ and Q^2^.

Variant	R^2^	Q^2^
PSC	0.640	0.535
MCC	0.467	0.364
MCB	0.610	0.484
PSC	0.556	0.427

**Table 7:** Table of path factors.

Trails	Path Factor (β)	STDEV	T	*p* Values	Valid
CO -> PSYC	0.377	0.035	10.684	0.000	YES
PEP -> PSYC	0.050	0.032	1.547	0.122	NO
SE -> PSYC	0.338	0.034	9.876	0.000	YES
PCR -> PSYC	0.218	0.037	5.910	0.000	YES
CO -> PSIC	0.010	0.024	0.414	0.679	NO
PEP -> PSIC	0.018	0.031	0.598	0.550	NO
SE -> PSIC	0.518	0.035	14.616	0.000	YES
PCR -> PSIC	0.429	0.034	12.462	0.000	YES
PSYC -> MCC	0.654	0.048	13.551	0.000	YES
PSIC -> MCC	0.056	0.045	1.233	0.218	NO
MCC -> MCB	0.615	0.039	15.626	0.000	YES
USC -> MCB	−0.280	0.041	6.787	0.000	YES
USC × MCC -> MCB	−0.187	0.040	4.697	0.000	YES

**Table 8:** Verification results of hypotheses.

Hypothesis	Content	Result
Hypothesis 1 (H1)	Consumer orientation has a positive impact on symbolic consumption perception and simulacrum consumption perception.	Invalid
Hypothesis 2 (H2)	Perceived economic stress positively affects symbolic consumption perception and simulacrum consumption perception.	Invalid
Hypothesis 3 (H3)	Self-expression positively affects symbolic consumption perception and simulacrum consumption perception.	Valid
Hypothesis 4 (H4)	Perceived consumption risk positively affects symbolic consumption perception and simulacrum consumption perception.	Valid
Hypothesis 5 (H5)	Symbolic consumption perception has a positive impact on minimalist consumption awareness.	Valid
Hypothesis 6 (H6)	Simulacrum consumption perception has a positive impact on minimalist consumption awareness.	Invalid
Hypothesis 7 (H7)	Minimalist consumption awareness positively influences minimalist consumer behavior.	Valid
Hypothesis 8 (H8)	Upward social comparison negatively moderates the influence of minimalist consumption awareness on minimalist consumer behavior.	Valid

## Data Availability

The original contributions presented in the study are included in the article, further inquiries can be directed to the corresponding author.
